# Developing and validating a nomogram for cognitive impairment in the older people based on the NHANES

**DOI:** 10.3389/fnins.2023.1195570

**Published:** 2023-08-17

**Authors:** Xiaoming Ma, Wendie Huang, Lijuan Lu, Hanqing Li, Jiahao Ding, Shiying Sheng, Meng Liu, Jie Yuan

**Affiliations:** ^1^North China University of Science and Technology, Tangshan, Hebei, China; ^2^Department of Neurology, The Third Affiliated Hospital of Soochow University, Changzhou, Jiangsu, China; ^3^Jitang College, North China University of Science and Technology, Tangshan, Hebei, China; ^4^Institution of Mental Health, North China University of Science and Technology, Tangshan, Hebei, China

**Keywords:** cognition disorders, aging, NHANES, ROC, prediction model

## Abstract

**Objective:**

To use the United States National Health and Nutrition Examination Study (NHANES) to develop and validate a risk-prediction nomogram for cognitive impairment in people aged over 60 years.

**Methods:**

A total of 2,802 participants (aged ≥ 60 years) from NHANES were analyzed. The least absolute shrinkage and selection operator (LASSO) regression model and multivariable logistic regression analysis were used for variable selection and model development. ROC-AUC, calibration curve, and decision curve analysis (DCA) were used to evaluate the nomogram’s performance.

**Results:**

The nomogram included five predictors, namely sex, moderate activity, taste problem, age, and education. It demonstrated satisfying discrimination with a AUC of 0.744 (95% confidence interval, 0.696–0.791). The nomogram was well-calibrated according to the calibration curve. The DCA demonstrated that the nomogram was clinically useful.

**Conclusion:**

The risk-prediction nomogram for cognitive impairment in people aged over 60 years was effective. All predictors included in this nomogram can be easily accessed from its’ user.

## 1. Introduction

An increasingly aging population means a higher incidence of aging-related disorders (Klimova and Maresova, 2017). Cognitive impairment is a part of the aging process (Rabin et al., 2018), which is characterized by a gradual deterioration of cognitive abilities in multiple domains, including memory and at least one additional area, such as learning, orientation, language, comprehension, and judgment (Daviglus et al., 2010). Though cognitive impairment is insufficiently severe to be diagnosed as dementia, it is still different from normal aging, which interferes with daily life. As an intermediate state between dementia and normal cognition, cognitive impairment preserves functional abilities (Hugo and Ganguli, 2014).

In the last decade, much effort has been made toward the early identification of cognitive impairment. Previous researchers have found associations among cognitive health, level of education (Yuan et al., 2018), and physical activity (Brown et al., 2021). Considering that cognitive impairment may be the prodrome of dementia, early identification and intervention may help slow down the process of cognitive decline (Lebedeva et al., 2015). Thus, developing a cognitive impairment prediction model to help the older people identify their own risk of developing cognitive impairment not only would lighten the patients’ burden but also improve their quality of life.

The NHANES is a series of cross-sectional surveys conducted by the Centers for Disease Control and Prevention (CDC) on a nationally representative population to provide health and nutrition data. The study participants were interviewed in their homes for the collection of demographic information. Subsequently, they visited a mobile examination center (MEC) for the collection of other data, including cognition tests. So far, many researchers (Li et al., 2022; Yang C. et al., 2022; Yang H. et al., 2022) have used the NHANES database to establish nomogram prediction models and achieved positive results. Therefore, we accessed it to conduct this research.

Previous studies identified the common risk factors. Diabetes mellitus can significantly increase the incidence of mild cognitive impairment as well as dementia (Biessels and Despa, 2018). Physical activity benefits cognition, especially executive functioning and memory (Nuzum et al., 2020). Males have a higher risk of cognitive impairment (Fu and Liu, 2022). A poorer education level is an independent risk factor for cognitive impairment (Wang et al., 2020). Hence, this study attempts to establish a predictive nomogram for cognitive impairment according to the social demographic characteristics, medical history, education level, and physical activity of the people aged above 60 years.

Though previous efforts have been made to develop models for cognitive impairment, some of them are complex and involve genetic sequencing (You et al., 2022) or are based on single-center retrospective studies (Zhou et al., 2021), requiring further validation of their clinical effectiveness. The purpose of our study is to develop a highly feasible predictive model for cognitive impairment using large-scale data from NHANES. Through this study, a nomogram was developed to predict the incidence of cognitive impairment in people over 60 years of age. It can help in the early detection of the risk of cognitive impairment in the older people and allow them to undergo further examination to adopt early intervention and even reduce the incidence of dementia.

## 2. Materials and methods

### 2.1. Study population and data

**Figure 1:**
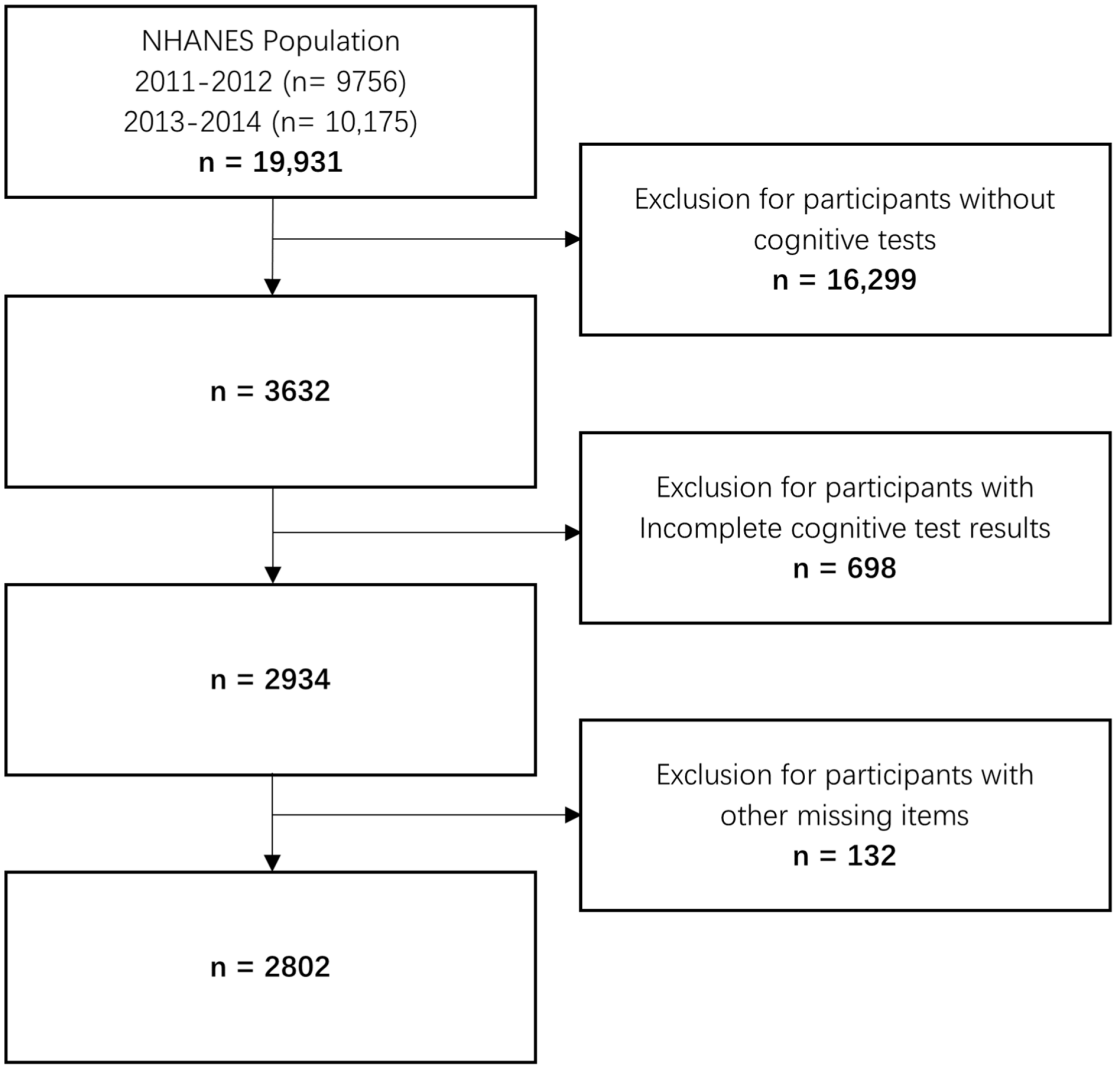
Flowchart of study participants.

The NHANES is a national cross-sectional study in the United States. It can be accessed through the Centers for Disease Control and Prevention National Center for Health Statistics (NCHS; https://www.cdc.gov/nchs/). The study protocol (Protocol #2011–17; Continuation of Protocol #2011–17) was approved by the NCHS Research Ethics Review Board, and all participants provided written informed consent before participation. Data from 2011–2012 to 2013–2014 were combined to perform the research. Demographic and questionnaire data were collected. People aged above 60 years and who had completed the cognitive function tests were included. The exclusion criterion was missing data in the items selected from the questionnaire dataset. Figure 1 shows the data processing details.

### 2.2. Cognitive function assessment

In the 2011–2014 NHANES study, the cognitive test was conducted in the MEC for people aged 60 years and above. The test consisted of four parts, the immediate recall test (IRT), the animal fluency test (AFT), the digital symbol substitution test (DSST), and the delayed recall test (DRT).

The IRT and DRT were used to assess immediate and delayed learning ability. In IRT, the participants were instructed to read 10 unrelated words each time and then recall as many words as possible. The DRT was set approximately 10 min after the start of the IRT, and the participants were asked to recall as many words as possible.

The AFT was used to test verbal category fluency, which reflected executive function status. The participants were asked to name as many animals as possible in 1 min. The AFT was a commonly used method to screen for cognitive impairment. For example, the United States Alzheimer’s Disease Joint Registration Cooperative Organization developed a set of 10 sub-tests in the late 1980s, 1 of which is the AFT (Morris et al., 1989).

The DSST as a performance module from the Wechsler Adult Intelligence Scale (WAIS III; Chen et al., 2017), was an instrument of processing speed, visual scanning, sustained attention, and working memory. The participants were provided with a piece of paper with nine numbers and corresponding symbols. They were asked to pair the symbols with 133 numbers within 2 min.

### 2.3. Definition of cognitive impairment

The definition of cognitive impairment used in this study was based on a previous study (Shi et al., 2023). Each test was further analyzed by calculating the z-score and then accumulated to a total score of cognition. The lowest quartile of the total score of cognition was used as the cutoff point, which was −2.12. The participants above the cutoff point were assigned to the Control Group; the others were assigned to the Cognitive Impairment Group.

### 2.4. Statistical analysis

Data analyses were applied using R software version 4.2.2. Non-normally distributed continuous variables were presented as the median, and categorical variables were presented as the number of cases (*n*) and frequency (%). The ANOVA test and chi-squared test were applied for comparing the differences between the groups. Since the R package for rms does not have a weighting procedure, we did not use the NHANES survey wights in our study. The least absolute shrinkage and selection operator (LASSO; Tibshirani, 1996) and logistic regression were used to examine the association between the cognitive test and other variables. All statistical tests were two-sided. The significance level was 0.05.

Meanwhile, the dataset was divided into training and validation sets in a 4: 1 ratio. The validation set was utilized to generate calibration curves to assess the model’s generalization capability. We utilized the “glmnet” package (version 4.1-4) to fit the LASSO regression, which can choose variables from a large and potentially multicollinear set of variables. A 10-fold cross-validation of the lambda value was conducted; non-zero coefficient variables were chosen to develop the multivariable logistic regression on the training set (Wen et al., 2019). Subsequently, the logistic regression was steamlined using the backward stepwise regression method.

Model performance was assessed using three recommended measures, the C-statistic (Caetano et al., 2018), the calibration curve (Van Calster et al., 2019), and the decision curve (Van Calster et al., 2018). The C-statistic is also known as the area under the receiver operating characteristic (ROC) curve, which measures the model’s ability to distinguish between patients with high or low risks. The calibration curve was plotted to show the relationship between predicted and observed outcomes in the dataset. The decision curve analysis (DCA) was applied to evaluate the net benefit of this prediction model, which is determined by calculating the difference between the expected benefit and expected harm in each proposed testing and treatment strategy. The threshold probability can be a certain level for appropriate intervention in clinical use.

## 3. Results

### 3.1. Characteristics of participants

In this study, there were 2,802 participants. Table 1 presents their demographic characteristics. Table 2 shows data according to the full cognitive test quartiles. The mean age was 69.41 ± 6.76 years, and 48.5% of the population was male. The two groups statistically differed in terms of age, gender, education level, marital status, history of hypertension, diabetes status, alcohol consumption status, moderate-intensity physical activity, and taste disturbance. After random grouping, there are no statistically significant differences observed between the training and validation groups.

**Table 1:** Demographic and cognitive characteristics of the study population (*n* = 2,802).

Characteristic	CI (*n* = 701)	NCI (*n* = 2,101)	All (*n* = 2,802)	*p*-value
Age (years)	73 (66–80)	67 (63–73)	68 (63–75)	<0.001
Minutes sedentary activity (minutes)	392.46 ± 200.23	395.49 ± 188.30	394.73 ± 191.32	0.717
Male (%)	381 (56.44%)	978 (45.98%)	1,359 (48.50%)	<0.001
Education (%)				<0.001
Less than 9th Grade	183 (27.11%)	121 (5.69%)	304 (10.85%)	
9-11th Grade	153 (21.83%)	240 (11.42%)	393 (14.03%)	
High school graduate	168 (23.97%)	488 (23.23%)	656 (23.41%)	
Some college or AA degree	112 (15.98%)	692 (32.94%)	804 (28.69%)	
College graduate or higher	80 (11.41%)	565 (26.89%)	645 (23.02%)	
Marriage (%)				<0.001
Married (include Separated)	384 (54.78%)	1,235 (58.78%)	1,619 (57.78%)	
Unmarried (including never married; divorced; widowed; living with partner)	317 (45.22%)	866 (41.22%)	1,183 (42.22%)	
Hypertension (Yes; %)	474 (67.62%)	1,279 (60.88%)	1753 (62.56%)	0.002
Dyslipidemia (Yes; %)	378 (53.92%)	1,200 (57.12%)	1,578 (56.32%)	0.152
Diabetes mellitus (%)				<0.001
Yes	201 (28.67%)	443 (21.09%)	644 (22.98%)	
Borderline	33 (4.71%)	93 (4.43%)	126 (4.50%)	
No	467 (66.62%)	1,565 (74.49%)	2032 (72.52%)	
Drink (Yes; %)	442 (63.05%)	1,472 (70.06%)	1914 (68.31%)	0.001
Walk/cycle (Yes; %)	136 (19.40%)	446 (21.23%)	582 (20.77%)	0.328
Moderate activity (Yes; %)	133 (18.97%)	643 (30.60%)	776 (27.69%)	<0.001
Sleep disorder (Yes; %)	74 (10.56%)	259 (12.33%)	333 (11.88%)	0.235
Smell alteration (%)				0.988
Better now	36 (5.14%)	111 (5.28%)	147 (5.25%)	
Worse now	122 (17.40%)	364 (17.33%)	486 (17.34%)	
No change	543 (77.46%)	1,626 (77.39%)	2,169 (77.41%)	
Taste alteration (Yes; %)	635 (90.58%)	1923 (91.53%)	2,558 (91.29%)	0.491
Smell problem (Yes; %)	76 (10.84%)	200 (9.52%)	276 (9.85%)	0.345
Taste problem (Yes; %)	57 (8.13%)	110 (5.24%)	110 (5.24%)	0.007

CI, cognitive impairment; NCI, non-cognitive impairment; Drink, had at least 12 alcoholic drinks per year; Minutes Sedentary Activity, minutes usually spend on a typical day.

**Table 2:** Univariable and multivariable logistic regression of predictors for cognitive impairment patients.

Variable	Univariate analysis			Multivariate analysis		
	OR	95% CI	*p*-value	OR	95% CI	*p*-value
Moderate activity						
No	1.00			1.00		
Yes	0.51	0.40–0.65	<0.001	0.60	0.46–0.78	<0.001
Taste Problem						
No	1.00			1.00		
Yes	1.77	1.23–2.56	0.002	1.87	1.25–2.81	0.003
Sex						
Female	1.00			1.00		
Male	1.66	1.37–2.01	<0.001	1.89	1.52–2.36	<0.001
Age, years	1.09	1.07–1.10	<0.001	1.10	1.08–1.12	<0.001
Education						
9-11th Grade (Includes 12th grade with no diploma)	1.00			1.00		
College Graduate or Higher	0.22	0.16–0.31	<0.001	0.19	0.13–0.26	<0.001
High School Graduate/GED or Equivalent	0.56	0.42–0.75	<0.001	0.52	0.39–0.69	<0.001
Less than 9th Grade	2.44	1.74–3.43	<0.001	2.59	1.87–3.59	<0.001
Some College or AA Degree	0.25	0.18–0.34	<0.001	0.25	0.19–0.34	<0.001

Moderate Activity: Have moderate-intensity activity causing small increases in breathing or heart rate, such as brisk walking or carrying a light load for at least 10 min at work.

### 3.2. Nomogram development and validation

**Figure 2:**
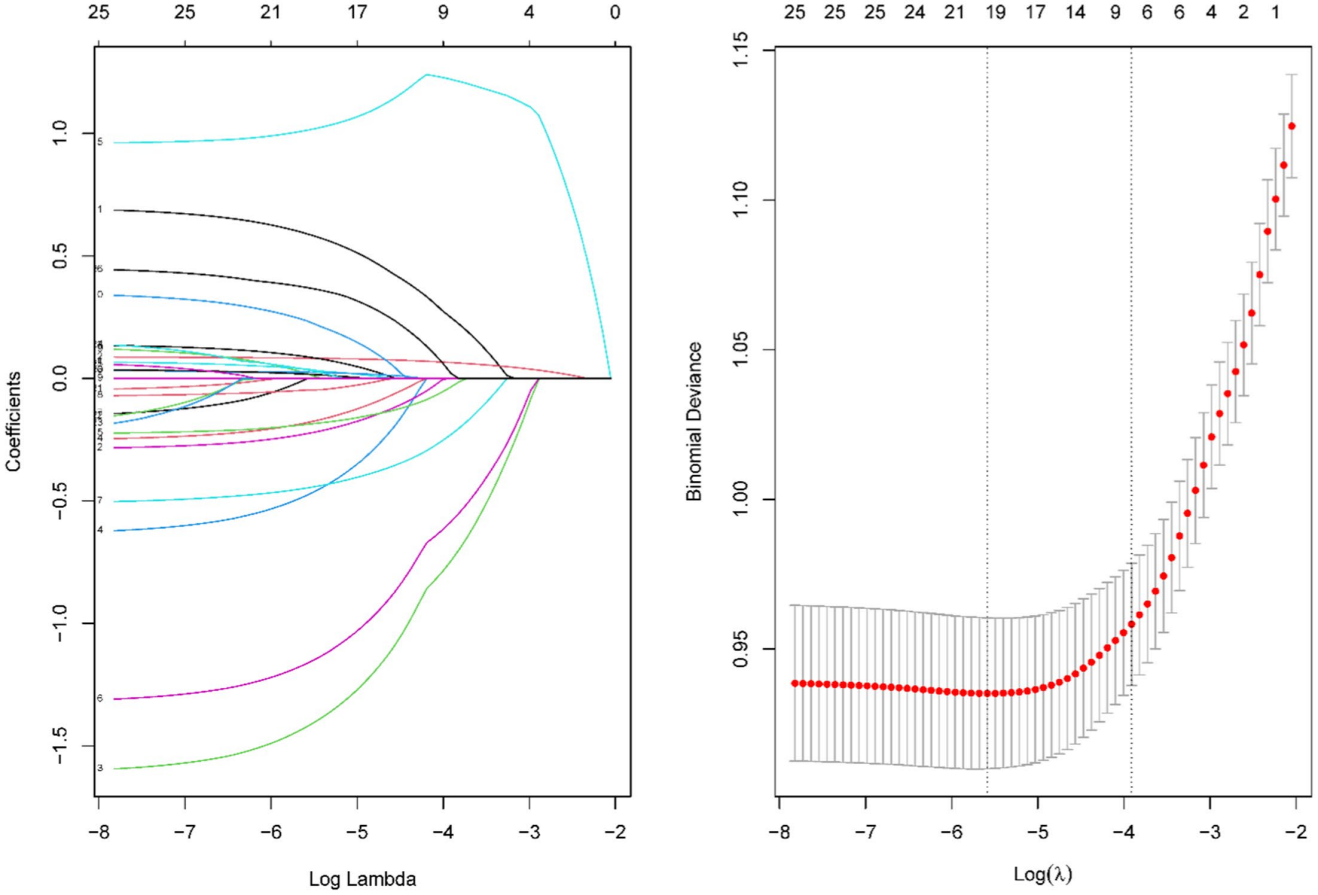
Results of the LASSO regression. Tuning parameter (λ) selection in the LASSO model using 10-fold cross-validation via minimum criteria.

**Figure 3:**
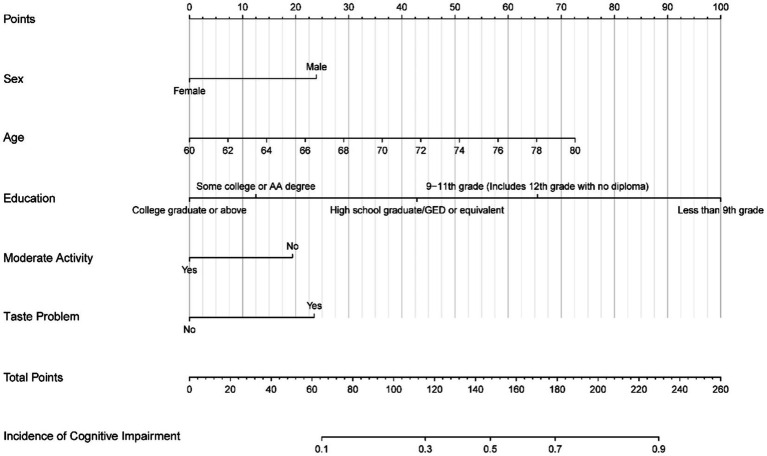
Nomogram predicting cognitive impairment among the older people.

According to the optimum λ value of the LASSO regression (Figure 2), six predictors were selected. A total of five variables were included in the logistic regression (Table 2), making the model with minimal Akaike information criterion (AIC) value, which means that the model had a better fit (Figure 3).

**Figure 4:**
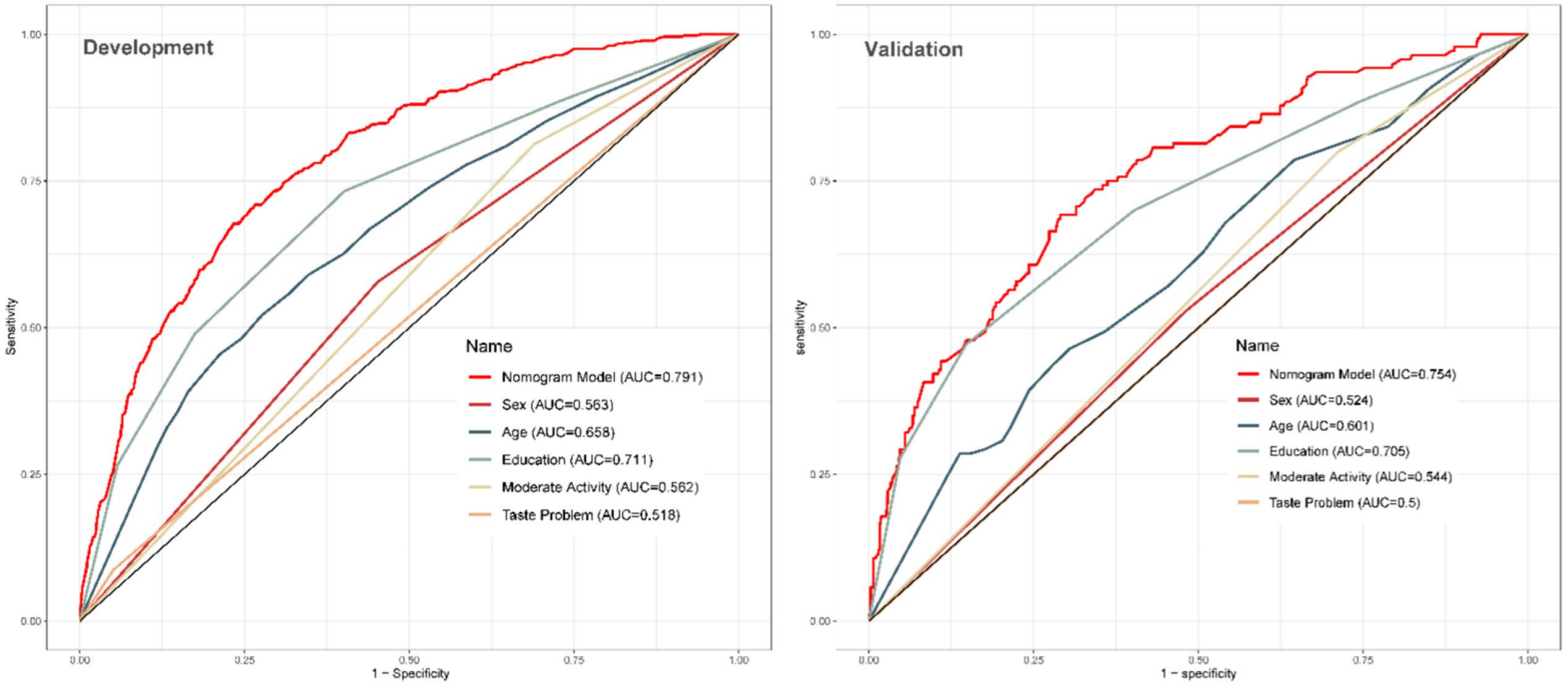
Analysis of ROC curve for the predictors. AUC, the area under the curve.

**Figure 5:**
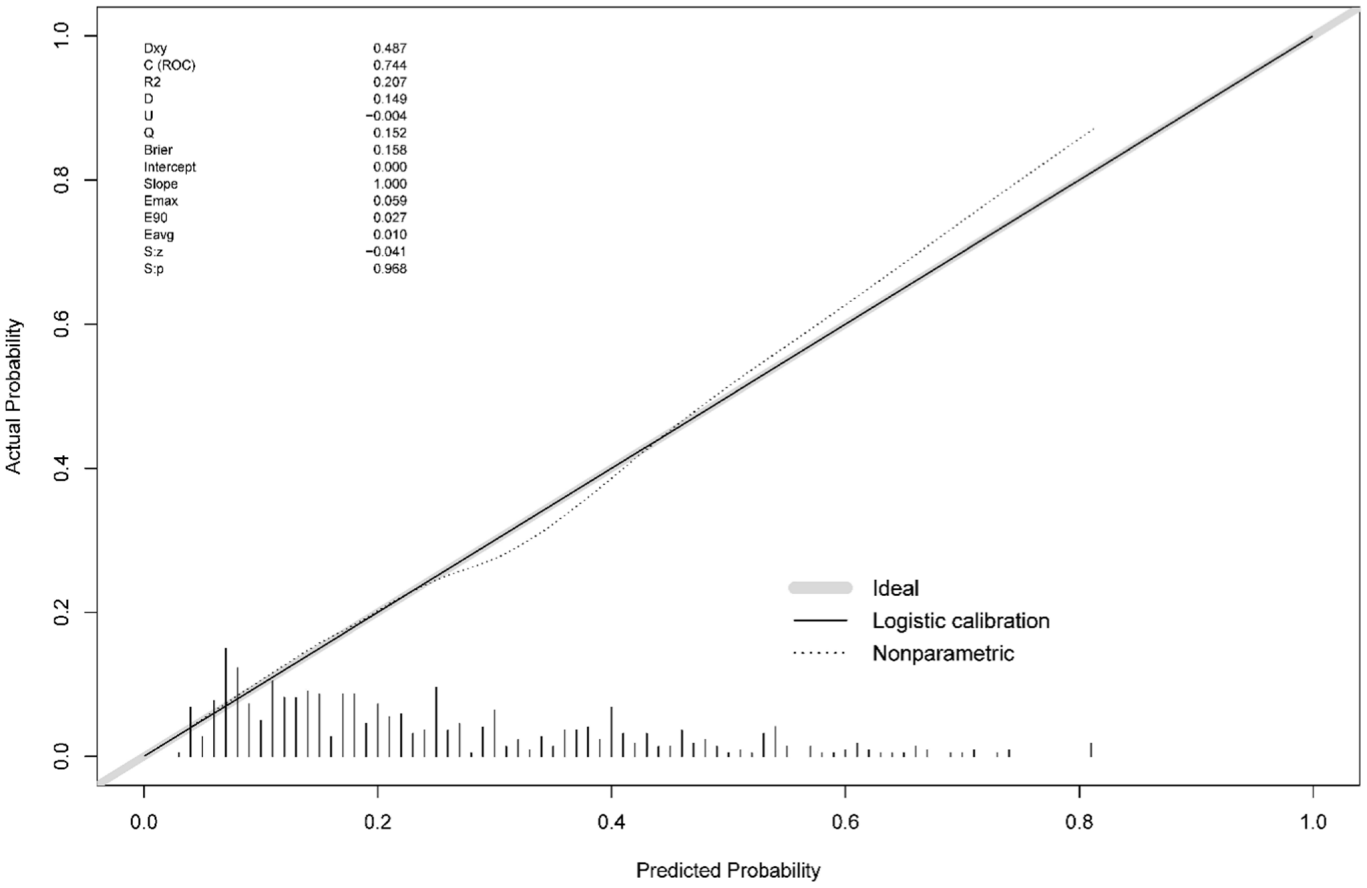
Calibration curves of the nomogram. The actual outcome rate is plotted on the y-axis; the nomogram-predicted probability of the outcome is plotted on the x-axis.

Based on the final model, the nomogram was constructed for people aged above 60 years. The risk factors included diabetes mellitus, sex, age and taste problems. The protective factors included moderate psychical activity and high education level. The nomogram achieved a AUC of 0.782 (95% CI 0.723–0.801; Figure 4). A calibration curve also indicated good consistency between the prediction and observed outcomes (Figure 5). The nomogram model performed well in predicting cognitive impairment among the older people, without external validation.

### 3.3. Clinical practice

**Figure 6:**
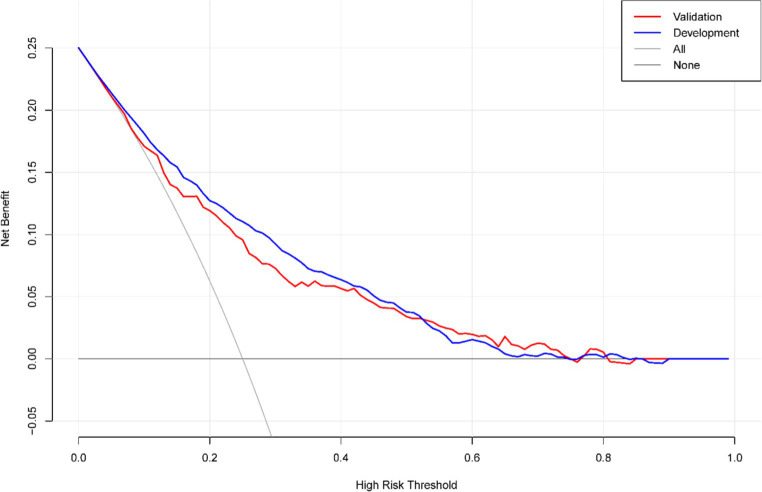
Decision curve assessment for the nomogram. In DCA, the nomogram shows more net benefits than full or no treatment across a threshold probability range.

The DCA for the nomogram was conducted to measure the risk and benefits. In Figure 6, the black horizontal axis means that no one received an intervention, and the net benefit is 0. The grey line means that all people received an intervention. According to the decision curve, the threshold probability >5% for the patient and <75% for the clinicians would benefit more from using this nomogram.

## 4. Discussion

This study developed and validated a prognostic nomogram based on a cross-sectional study from NHANES (2011–2014) to predict the probability of cognitive impairment in people above 60 years of age. The nomogram included five variables, each of which can be easily acquired from its’ user.

Our study findings are consistent with evidence from a previous study (Lövdén et al., 2020). Among the five independent predictors, education level contributed the most to predicting the outcome. It had been widely accepted that education plays an important role in the decline of cognitive functions. People with a higher education level are less likely to experience a decline in cognitive function. They also experience a slower rate of cognitive decline regardless of neurodegenerative or vascular pathologies (Members et al., 2010). Therefore, improving the education conditions during the initial decades of life and prolonging the educational years are crucial to reducing the cognitive impairment of the older people (Lövdén et al., 2020).

Table 2 shows the significant difference between the two groups in terms of moderate-intensity physical activity. Although there was no statistical difference in sedentary time between the two groups, there was a significant difference in cognitive function due to the difference in moderate activity time. Notably, physical activity was a direct and feasible variable among the five variables and had been proven as being highly related to better cognitive function in old age (Reas et al., 2019). In an umbrella review conducted by the 2018 Health and Human Services Physical Activity Guidelines for Americans Advisory Committee, Erickson et al. analyzed large amounts of data from randomized controlled trials to prove that moderate-intensity physical activity is associated with cognitive improvement (Erickson et al., 2019). They also found strong evidence proving that higher physical activity is associated with a reduced risk of developing cognitive impairment, including Alzheimer’s disease. Thus, increasing moderate-intensity physical activity may be an effective and quick way to improve cognitive function among the older people, and many experts agree with this (Bangsbo et al., 2019).

Taste plays a crucial role in individual assessment of the nutritional value, safety, and quality of food. Although both olfaction and taste tend to decline with age, research (Ogawa et al., 2017) has shown that older adults who experience taste problems or a decline in taste sensitivity often exhibit earlier cognitive function decline.

Although a study (Hu et al., 2019) also using the NHANES database to discuss cognitive impairment showed that moderate to severe depressive symptoms are associated with poorer cognitive function in the older people and more so in the case of women than men. This is contrary to our study wherein being male proved to be a risk factor for cognitive impairment. The explanation for the controversy is that Hu’s study focuses on the association between depression, cognitive function, and gender, rather than exploring any potential factors related to cognitive decline in the general elderly population, as done in our study. Due to the population restriction of Hu’s study to the elderly depression group, it’s not appropriate to compare the variable” gender” at the same level of influencing factors between the two articles. Additionally, many studies explored the relationship between gender and cognitive impairment and eventually obtained different answers. Consistent with our conclusion, Petersen et al. found that the prevalence of mild cognitive impairment is higher in men (Petersen et al., 2010). The relationship between gender and cognitive impairment is still inconclusive. Conducting more studies in this regard may yield clear conclusions.

As a non-intervention factor, age plays an important role in cognitive impairment. Cognitive impairment is increasingly common in the process of aging (Plassman et al., 2008), and the prevalence of mild cognitive impairment increases with age (Li et al., 2020). Hence, the older population experiences a higher incidence of cognitive impairment. That is why we focused on studying and building a nomogram for cognitive impairment for the older people.

Our nomogram integrated different prognostic variables and can generate an individual probability of cognitive impairment among the older people. It included age, sex, education level, taste problem and moderate activity. Each of these is easy to access from the user. Moreover, our nomogram showed good performance in the cohort, regardless of the discriminatory and calibration capacity. It is very convenient to use. For example, a 76-year-old male with no history of diabetes and no regular physical exercise and who graduated from high school when he was young, received a total score of 135 (0 for diabetes, 20 for moderate activity, 55 for age, and 39 for sex), indicating the predicted risk of cognitive impairment of about 69%. This case shows that the older people can easily complete the risk assessment and seek help or intervention on time. A larger cohort study is needed to further explore the five results proposed by our study. Other variables not included in the final regression model but which have the predictive value of statistical differences within the studied population should also be explored.

## 5. Conclusion

By conducting a thorough analysis of NHANES data from 2 years cycles, this study provides compelling evidence to validate the significant association between cognitive impairment in the older people and five key factors, namely sex, age, educational level, engagement in moderate-intensity physical activities in daily life, and the presence of taste problems. Our findings emphasize the importance of considering these factors for achieving accurate predictions of cognitive impairment among the older population.

## Data availability statement

The original contributions presented in the study are included in the article/Supplementary material, further inquiries can be directed to the corresponding authors.

## Author contributions

SS, ML, and JY came up with the idea and designed the study. XM and WH conducted statistical analysis and scientific writing. LL, HL, and JD wrote the manuscript. All authors contributed to the article and approved the submitted version.

## Conflict of interest

The authors declare that the research was conducted without any commercial of financial relationships that could be constructed as a potential conflict of interest.

## Publisher’s note

All claims expressed in this article are solely those of the authors and do not necessarily represent those of their affiliated organizations, or those of the publisher, the editors and the reviewers. Any product that may be evaluated in this article, or claim that may be made by its manufacturer, is not guaranteed or endorsed by the publisher.
