# Supplementary material for: Developing and validating a nomogram for cognitive impairment in the older people based on the NHANES
Source: Front Neurosci. 2023 Aug 17;17:1195570. doi: 10.3389/fnins.2023.1195570 (PMC10470068; doi:10.3389/fnins.2023.1195570)
Supplement: Supplementary file 2 [file Data_Sheet_2.docx]

Supplementary Material

Developing and Validating a Nomogram for Cognitive Impairment in the Elderly Based on the NHANES (2011-2014)

**Xiaoming Ma1†, Wendie Huang2†,Lijuan Lu2†, Hanqing Li2†, Jiahao Ding1†, Shiying Sheng2*, Meng Liu2*, Jie Yuan3***

^1^North China University of Science and Technology, Tangshan, Hebei, China

^2^Department of Neurology, The Third Affiliated Hospital of Soochow University, Changzhou, Jiangsu, China

^3^Institution of Mental Health, North China University of Science and Technology, Tangshan, Hebei, China

*** Correspondence:**Shiying Sheng
dr-shengshiying@163.com
Meng Liu
lm145@163.com
Jie Yuan
tsphyj@126.com

# Supplementary Figures and Tables

## Supplementary Tables

**Supplementary Table 1**

Single-factor logistic regression outcomes for all variables

| Variable | OR | 95% CI | *p*-value |
| --- | --- | --- | --- |
| Gender |  |  |  |
| Female | 1.00 |  |  |
| Male | 1.56 | 1.31-1.85 | <0.001 |
| Age, years | 1.08 | 1.07-1.1 | <0.001 |
| Education |  |  |  |
| 9-11th Grade (Includes 12th grade with no diploma) | 1.00 |  |  |
| College Graduate or Higher | 0.22 | 0.16-0.3 | <0.001 |
| High School Graduate/GED or Equivalent | 0.54 | 0.41-0.71 | <0.001 |
| Less than 9th Grade | 2.54 | 1.87-3.46 | <0.001 |
| Some College or AA Degree | 0.25 | 0.19-0.34 | <0.001 |
| Moderate Activity |  |  |  |
| No | 1.00 |  |  |
| Yes | 0.53 | 0.43-0.66 | <0.001 |
| Diabetes Mellitus |  |  |  |
| Borderline | 1.00 |  |  |
| No | 0.84 | 0.56-1.27 | 0.408 |
| Yes | 1.28 | 0.83-1.97 | 0.263 |
| Hypertension |  |  |  |
| No | 1.00 |  |  |
| Yes | 1.34 | 1.12-1.61 | <0.001 |
| Marriage |  |  |  |
| Divorced | 1.00 |  |  |
| Living with Partner | 1.1 | 0.6-2.02 | 0.748 |
| Married | 1.24 | 0.94-1.63 | 0.129 |
| Never Married | 1.29 | 0.83-2 | 0.257 |
| Separated | 2.7 | 1.58-4.61 | <0.001 |
| Widowed | 2.12 | 1.57-2.88 | <0.001 |
| Drink |  |  |  |
| No | 1.00 |  |  |
| Yes | 0.73 | 0.61-0.87 | <0.001 |
| Dyslipidemia |  |  |  |
| No | 1.00 |  |  |
| Yes | 0.88 | 0.74-1.04 | 0.140 |
| Walk/Cycle |  |  |  |
| No | 1.00 |  |  |
| Yes | 0.89 | 0.72-1.11 | 0.299 |
| Sit | 0.99 | 0.99-1.00 | 0.716 |
| Sleep Hours | 1.05 | 0.99-1.11 | 0.135 |
| Sleep Disorder |  |  |  |
| No | 1.00 |  |  |
| Yes | 0.84 | 0.64-1.1 | 0.204 |
| Smell Alteration |  |  |  |
| Better Now | 1.00 |  |  |
| No Change | 1.03 | 0.70-1.52 | 0.883 |
| Worse Now | 1.03 | 0.67-1.59 | 0.880 |
| Taste Alteration |  |  |  |
| No | 1.00 |  |  |
| Yes | 0.89 | 0.66-1.2 | 0.447 |
| Smell Problem |  |  |  |
| No | 1.00 |  |  |
| Yes | 1.16 | 0.87-1.53 | 0.314 |
| Taste Problem |  |  |  |
| No | 1.00 |  |  |
| Yes | 1.60 | 1.15-2.23 | 0.007 |

**Supplementary Table 2**

Survey-multivariable logistic regression

| Character | OR | 95% CI | *p*-value |
| --- | --- | --- | --- |
| Diabetes Mellitus |  |  |  |
| Borderline | 1.00 |  |  |
| No | 1.11 | 0.55-2.24 | 0.76 |
| Yes | 1.64 | 0.74-3.66 | 0.21 |
| Moderate Activity |  |  |  |
| No | 1.00 |  |  |
| Yes | 0.57 | 0.42-0.78 | 0.001 |
| Gender |  |  |  |
| Female | 1.00 |  |  |
| Male | 1.46 | 1.09-1.96 | 0.01 |
| Age, years | 1.12 | 1.09-1.15 | <0.0001 |
| Education |  |  |  |
| 9-11th Grade (Includes 12th grade with no diploma) | 1.00 |  |  |
| College Graduate or Higher | 0.21 | 0.14-0.31 | <0.0001 |
| High School Graduate/GED or Equivalent | 0.74 | 0.52-1.07 | 0.11 |
| Less than 9th Grade | 3.14 | 1.87-5.27 | <0.001 |
| Some College or AA Degree | 0.31 | 0.21-0.47 | <0.0001 |
